# Editorial: The Tumor Microenvironment: Recent Advances and Novel Therapeutic Approaches

**DOI:** 10.3389/fcell.2020.586176

**Published:** 2020-09-17

**Authors:** Hasan Korkaya, Sandra Orsulic

**Affiliations:** ^1^Department of Biochemistry and Molecular Biology, Georgia Cancer Center, Medical College of Georgia, Augusta University, Augusta, GA, United States; ^2^UCLA David Geffen School of Medicine, University of California, Los Angeles, Los Angeles, CA, United States

**Keywords:** tumor microenvironment, ECM, TAM, CAF, MDSC, CSC

Tumor cells establish a complex ecosystem called the tumor microenvironment (TME) which consists of stromal cells, immune cells, extracellular matrix (ECM) macromolecules and enzymes (Figure 1). It is now well-established that TME of solid tumors plays a fundamental role in tumor progression and metastasis, determining the disease outcome. In an age of molecularly targeted therapeutics and immune check point inhibitors (ICI), the role of TME in therapeutic resistance has become a major research focus. Although ICIs exhibited remarkable long-lasting responses in hard to treat malignancies, such as non-small cell lung cancer, renal cell carcinoma and melanoma (Nixon et al., 2018), these inhibitors alone or in combination with chemotherapy have shown very little promise in other solid tumors, such as breast, prostate and brain (Chai et al., 2019). Clinical studies revealed that tumor-infiltrating lymphocytes (TILs) are associated with the earlier stages of tumor progression and good prognosis in triple-negative breast cancer (Adams et al., 2014). However, the tumor-infiltrating myeloid precursors (tumor associated macrophages-TAMs, myeloid derived suppressor cells-MDSCs, regulatory dendritic cells-DCs and neutrophils) and cancer associated fibroblasts (CAFs) establish an immunosuppressive TME which is associated with advanced tumor stage and therapeutic resistance (Munn and Bronte, 2016). In addition, tumors establish their own vasculature via secreting angiogenic factors which in turn chemoattracts the endothelial cells within the tumor microenvironment. Crosstalk between the endothelial and tumor cells has been shown to play a major role in tumor progression and metastasis as well as resistance to a wide range of therapeutics including ICIs. Although pericytes were originally identified to surround and physically support new blood vessels, their critical importance in the premetastatic niche has recently been recognized. Recent data suggested that pericytes regulate immune responses by secreting adhesion molecules and a wide range of chemokines (Paiva et al., 2018). While angiogenesis inhibitors (AI) have been approved for the treatment of some solid tumors, currently there are several early clinical trials testing the efficacy of AIs in combination with ICIs (Ciciola et al., 2020). Furthermore, tumor-infiltrated myeloid precursors and fibroblasts regulate tumor cell plasticity and stemness during the metastatic cascade (Ouzounova et al., 2017). Malignant cells induce a dynamic stromal reaction, which both resists and promotes tumor progression. Additionally, ECM secreted by stromal cells play a crucial role in tumor progression and metastasis as well as therapeutic resistance.

**Figure 1:**
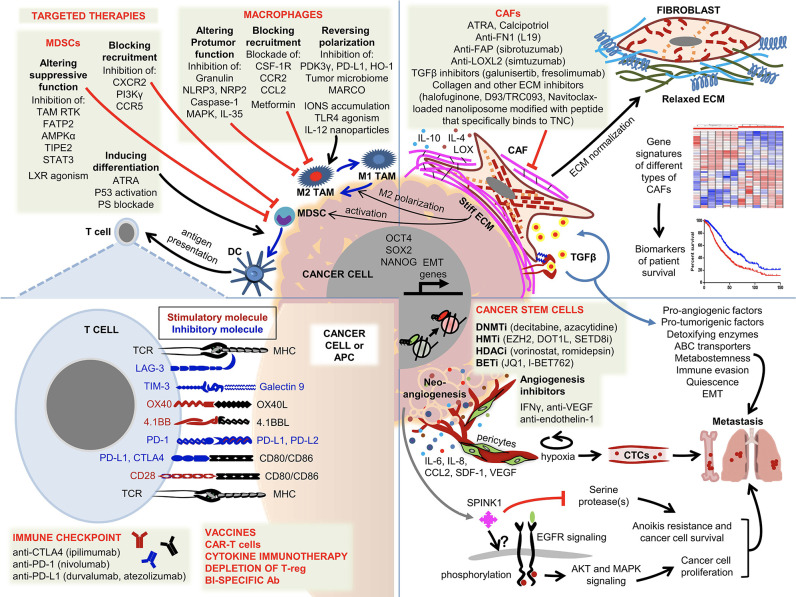
Overview of the components of the tumor microenvironment and current strategies to target these cells. Malignant cells establish early cross-communications with a wide range of host cells forming a favorable microenvironment consisting of fibroblasts, immune cells, ECM, endothelial cells and pericytes. While infiltration of T lymphocytes is a good prognostic factor, the majority of invasive/metastatic carcinomas establish immunosuppressive and tumor-promoting TMEs. The complex interaction between the tumor cells and the other components within the TME is illustrated and the following abbreviations are used. ABC transporter, ATP binding cassette transporter; Ac, acetylation; APC, antigen presenting cell; ATRA, all trans retinoic acid; BETi, bromodomain inhibitors; CAF, cancer-associated fibroblast; CAR-T-cell, chimeric antigen receptor T cell; CTC, circulating tumor cell; DC, dendritic cell; DNMTi, DNA methyltransferase inhibitors; ECM, extracellular matrix; EMT, epithelial-mesenchymal transition; HDACi, histone deacetylase inhibitors; HMTi, histone methyltransferase inhibitors; MDSC, myeloid-derived suppressor cell; Me, methylation; MHC, major histocompatibility complex; TAM, tumor associated macrophage; TCR, T-cell antigen receptor; Treg, regulatory T cell.

The following articles provide a comprehensive review of the current understanding of TME as illustrated in Figure 1. Ireland and Mielgo reviewed the literature on the roles of macrophages and fibroblasts. The authors first examined the origin of these cells and their physiological functions and discussed how they are activated in the context of malignancies. Unlike their quiescent counterparts, CAFs are activated in response to chronic tissue damage which is also called “wounds that do not heal,” leading to crosstalk between tumor cells and CAFs via a wide range of cytokines. Among these cytokines, TGFβ and IL6 play a major role in therapeutic resistance by driving an epithelial mesenchymal transition (EMT) in tumor cells. Tissue resident or bone marrow (BM) derived macrophages are recruited to, and activated at, the tumor site by tumor-derived factors. Upon failing to eradicate tumors, M1-like macrophages are polarized to M2 TAMs by tumor-secreted immunosuppressive cytokines, such as TGFβ, IL4 and IL10. The authors also discuss current efforts to target CAFs and M2 TAMs with therapeutic approaches ranging from blocking the recruitment of macrophages to the tumor site to repolarizing them into M1-like macrophages and reprogramming CAFs. Ongoing phase I/II clinical trials include the blockade of CSF1, CSF1R, CCL2, and CD40 in M2 TAMs and Smo and IGF in CAFs. Similarly, Liu et al. discussed how fibroblasts build the tumor microenvironment by secreting ECM, associated enzymes and other factors. The authors reviewed the recent literature on the critical importance of CAF-ECM and CAF-tumor cell interactions in tumor cell migration and invasion. LOX-induced ECM crosslinking has emerged as a viable molecular target and several LOX inhibitors are in preclinical development. Furthermore, Nallanthighal et al. provided a comprehensive review on the mechanistic properties of ECM that regulate the phenotype of cancer stem cells (CSC). Tumor ECM is stiffer due to overexpression of a wide range of macromolecules and ECM-modifying enzymes, which causes transmission of the signal to CSCs via focal adhesion kinase (FAK) and its downstream effectors driving the transcription of stemness genes, such as OCT4, SOX2, and NANOG. CSCs also evade immune recognition by ECM mediated activation of the PI3K/Akt pathway.

In contrast to solid tumors, Haro and Orsulic reported a favorable survival outcome that is associated with CAFs in B-cell lymphoma patients. Potential mechanisms proposed are the entrapment of malignant B cells by CAFs and associated ECM in lymph nodes and induction of apoptotic cell death by CAF-derived TGFβ.

In Turdo et al., the authors discussed potential strategies to target CSCs by blocking the stem cell specific pathways such as Notch, Wnt, and Hedgehog or TME-produced cytokines that regulate the stemness of malignant cells. Efforts to sensitize CSCs to ICIs by either CSC-primed dendritic cells or CAR T cell therapy with CSC-specific antigens were also discussed.

One potential target in TME are the immune cells of myeloid origin. Chaib et al. discussed the types of myeloid cells, their significance in tumor progression and recent strategies to target this cell population. Due to their suppressive activity, MDSCs have been implicated in the development of therapeutic resistance against ICIs. The authors discussed a comprehensive list of myeloid cell targets, such as PI3Kγ, NF-κB, CSF, CCL2, TLRs and histone deacetylases in pre-clinical and early clinical trials. A comprehensive review by Canning et al. examined the immune landscape of head and neck squamous cell carcinomas (HNSCC) according to human papilloma virus (HPV) status. While HPV(+) HNSCCs were infiltrated with lymphocytes that correlated with better clinical outcome, HPV(–) counterparts were infiltrated with highly immunosuppressive immune cells that correlated with poor outcome. In early clinical trials with ICIs, HPV(+) patients experienced better clinical outcomes compared to HPV(–) patients despite their high tumor mutation burden. These findings concur with the immunosuppressive role of TME in therapy response in solid tumors.

Finally, Mehner and Radisky reviewed the dual role of serine protease inhibitor Kazal type 1 (SPINK1) in the tumor and the TME. Aberrantly overexpressed SPINK1 in a wide range of solid tumors and the stroma renders tumor cells resistant to apoptotic cell death and increases cell proliferation, while enhanced expression in the tumor stroma contributes to therapeutic resistance.

In summary, although TME is viewed as a promising therapeutic target, we still face significant challenges in developing adequate tools to effectively target these cells. Current variable successes in targeting tumor stroma highlight the need to better understand the molecular characteristics of stromal cells to develop more precise and less toxic targeted therapies.

## Author Contributions

HK wrote the manuscript. SO edited the manuscript and drew the figure. All authors contributed to the article and approved the submitted version.

## Conflict of Interest

The authors declare that the research was conducted in the absence of any commercial or financial relationships that could be construed as a potential conflict of interest.
